# Case report on secondary testicular necrosis due to fulminant epididymitis: ultrasonographic evaluation and diagnosis

**DOI:** 10.1186/s12894-020-00655-w

**Published:** 2020-08-04

**Authors:** Wout Devlies, Mattias Seghers, Kurt Dilen

**Affiliations:** 1 Faculty of Medicine, KU Leuven, Leuven, Belgium; 2 Department of Urology, Regional hospital St. Franciskus, Heusden-Zolder, Belgium; 3 Department of Radiology, Regional hospital St. Franciskus, Heusden-Zolder, Belgium

**Keywords:** Testicular necrosis, Epididymitis, UTI, Complication

## Abstract

**Background:**

Scrotal pain is a common complaint in the clinical practice, with many underlying causes. Infectious causes, like epididymitis, are frequently encountered in the work-up of scrotal pain. The presentation of epididymitis is mostly mild, yet major complications can occur.

**Case presentation:**

We present a 35-year-old male presenting with scrotal pain and swelling of the testicle. Epididymitis with testicular necrosis was diagnosed using repeated doppler ultrasonography measurements. An orchiectomy was performed which showed a hemorrhagic process with affected spermatic cord. Funiculitis together with epididymal swelling can impede testicular blood flow, with testicular necrosis possibly resulting in orchiectomy. This is the first case that proved funiculitis to co-exist in loss of colour doppler on pathological evaluation.

**Conclusions:**

In order to reduce major complications, medical therapy should be promptly initiated when there is a suspicion of epididymitis.

## Background

Acute scrotal pain is frequently encountered at the emergency department. The differential diagnosis is broad, with sometimes difficult differentiation [1]. Different causes consist of acute epididymitis, testicular torsion, Fournier’s gangrene, torsion of appendix testis, trauma, post-vasectomy pain, inguinal hernia, mumps orchitis, testicular cancer, immunoglobulin A (IgA) vasculitis, and acute idiopathic scrotal edema. Due to the extensive list of diagnosis the standard of care is to identify causes that need immediate action. Testicular torsion and Fournier’s gangrene [2] are examples needing urgent surgical intervention. In acute epididymitis/ epididymo-orchitis medical treatment should start promptly, ultrasonography can help to prove the diagnosis and help with the prognosis of this infection [3].

Testicular torsion and major epididymo-orchitis can be difficult to distinguish. This distinction is made on blood results with infectious episodes resulting in higher C-reactive protein levels. One rare complication in epididymitis is testicular necrosis, with an incidence of 1–2% of epididymitis cases. In case of post-infectious necrosis, the presentation to surgery-time was significantly longer than in testicular torsion [4].

On ultrasonography epididymo-orchitis necrosis can be recognized by a juxta-epididymal string-of-bead sign, contrasting the whirlpool/knot sign, seen in testicular torsion [4]. Reduction of color doppler signalling with high intratesticular resistive indices and negative diastolic flow increase the likelihood of testicular necrosis associated with epididymo-orchitis [5, 6]. Further assessment of suspected testicular necrosis and its vascularity can be performed using ultrasound microbubble contrast [7].

Surgical management in necrosed testis is an orchiectomy. Fasciotomy in early hypoperfusion has been suggested in the past, but no clinical outcomes have been reported [8]. This is therefore not routinely done. Partial hypoperfusion and necrosis of the testis can be treated conservatively with close follow-up.

A Medline search was initiated to discuss similar cases and their treatment approaches using MeSH terms: testis, orchitis, epididymitis, necrosis and infection. There is currently a lack of data and clinical guidance in patients presenting with these complications. Together with the available literature, this case report can support clinicians in their daily practise.

## Case presentation

**Fig. 1:**
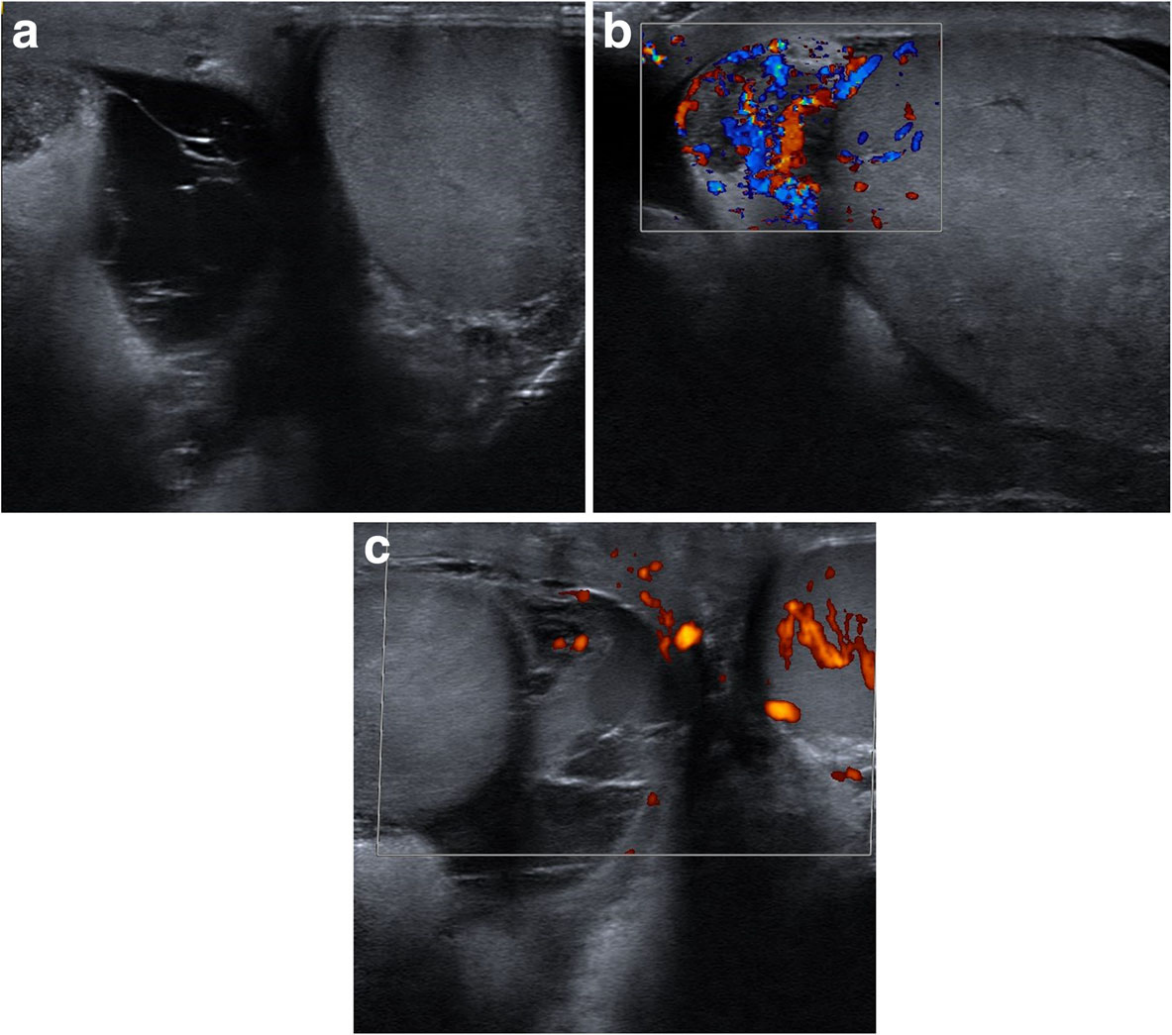
**a**-**b**: Initial ultrasonographical images with **a**: Pyocoele, **b**: accentuated doppler of epididymo-orchitis. **c**: Images of the second ultrasonographical evaluation, with right-sided avascular necrosis with pyocoele in comparison to a normal left testis

A 35-year old male, with no relevant past medical history, presented to the emergency room with local swelling, fever of 39.4 °C (103 °F) and pain in the right hemiscrotum. It started mildly a week earlier and he was started on ciprofloxacin 500 mg since 4 days. There was no history of trauma and no recent disease signs. A scrotal ultrasound was performed on admission showing an enlargement of the epididymis on the right side with septal hydrocoele (Fig. 1a). By using scrotal color doppler ultrasound scanning an increased signal was seen in the right testis (Fig. 1b). Blood results initially showed C-reactive protein (CRP) of 69 mg/L with a leucocytosis of 20,000/mm^3^ and left shift.

The diagnosis of epidymo-orchitis was made with a prompt start of IV antibiotics (ceftriaxone 2 g 1x/day with amikacin 1 g/day). On repeated bloods the next day, the CRP rose to 230 and 320 mg/L one and 2 days after presentation respectively with remaining leucocytosis of 29,000/mm^3^ and 22,000/mm^3^. Initial urine culture showed the presence of an extended spectrum beta lactamase *E. coli*, sensitive to ceftriaxone and temocillin, resistant to Fluoroquinolones. After knowledge of the antibiogram, the patient was put on temocillin.

Two days after the initial presentation, the symptomatology did not improve notably. A re-assessment with ultrasonography and blood results was done. This showed a stable hypervascularisation and swelling in the epididymis. The septal hydrocele enlarged in 2 days’ time with an appearance of heterogeneity in the hydrocele collection. Upon second ultrasound evaluation, no residual color Doppler signal was found in the testis (Fig. 1c).

Due to the evolving ultrasonographic findings and the loss of doppler signal an orchiectomy was performed. The testis was removed together with the epididymis and the existing pyocele. The operation field was thoroughly cleaned. Postoperative blood analysis showed a CRP level of 210 mg/L with a leucocytosis of 18,000/mm^3^.

**Fig. 2:**
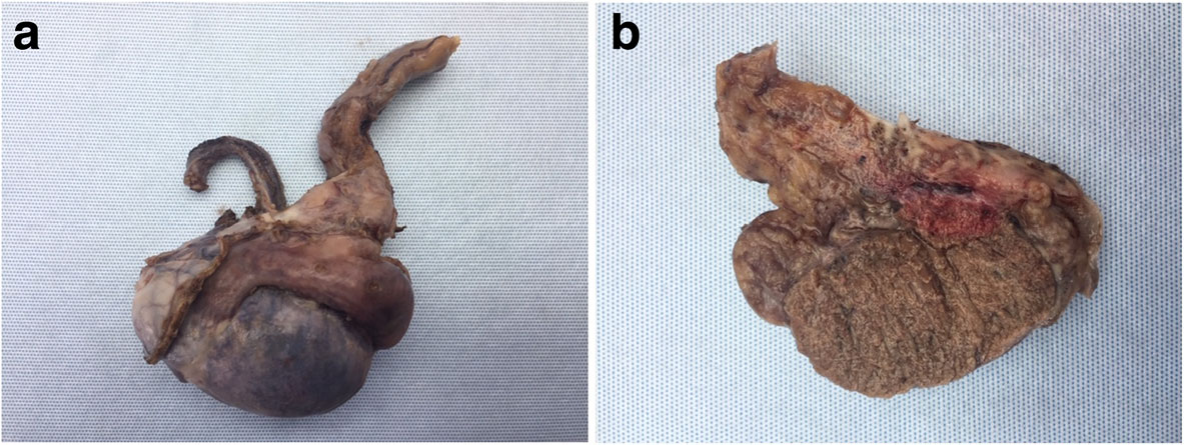
Postoperative resection specimen **a**: pathology specimen **b** cross-sectional section of the testis

On anatomopathological review these structures were primarily typed of hemorrhagic substrate with mixed inflammatory cells (Fig. 2). The spermatic cord in this resection is also taint in a similar process. No signs of malignancy are seen in the resection specimen. A microbiological examination of the orchiectomy specimen could confirm the presence of E Coli in the resection specimen, therefore providing additional evidence for an epidydimo-orchitis, yet this was not performed.

The patient went home with outpatient parenteral antimicrobial therapy of Temocillin for 7 days. The blood abnormalities gradually decreased with a reassuring clinical picture. The patient remained asymptomatic and afebrile since.

## Discussion

Testicular necrosis is a rare presentation of epididymitis in the emergency department, with an estimated incidence of 1–2% of all epididymitis cases [9, 10].

Our case highlights the benefit of ultrasonography of the testis. This case initially showed clear hyperperfusion with a rapid change to hypoperfusion. The latter being strongly associated with testicular necrosis [9].

Moreover, this paper proves to be a contemporary example of the approach in severe epididymitis and the useful ultrasonographical measures. As ultrasonography is frequently performed and relatively cheap, but on the other hand user dependent, this paper supplied us with evidence on how to diagnose similar conditions.

We preferred to surgically remove the testicle directly after loss of doppler signal, yet other authors have reported acceptable results with longer intervals between diagnosis and surgery [11]. When longer intervals are used a fistula would be formed which should then be surgically removed [12, 13].

Testicular necrosis is caused by venous occlusion from the testes [11, 14]. The mechanism of this para-infectious thrombosis is not entirely clear [11, 15]. Suggested mechanisms contributing to this testicular necrosis could be venous compression by oedema of the epididymis, swelling of the funiculus in case of funiculitis or edema at the external inguinal ring. Besides this, bacterial toxins may damage endothelial cells with induction of vascular thrombosis [15–17]. In rodents infected with E Coli, necrosis is found to be the primary cell death mechanism. This effect is primarily seen in Sertoli cells in infected testis with impaired spermatogenesis due to the lack of supporting germ cells [18]. This lowering of spermatogenesis after infectious necrosis is also seen in human testis [11].

As discussed different etiologies have been postulated with few proven cases, with few high quality evidence reported in literature. To our knowledge, this is the first case that proved funiculitis to co-exist in loss of colour doppler on pathological evaluation.

## Conclusion

Epididymitis is a common condition with possible severe complications. If epididymitis is suspected we suggest to start medical treatment promptly. In our experience, scrotal ultrasound should be performed for the diagnosis of epididymitis and orchitis and should be repeated if there is a suspicion of doubtful blood supply to the testicle. This case provides the first clinical evidence to support associated funiculitis in this disease. Also this case stresses the usefulness of ultrasonography in the diagnosis and management of testicular necrosis secondary to epididymitis/ epididymo-orchitis.

## Data Availability

All data generated or analysed during this study are included in this published article.

## Abbreviations

MeSH: Medical subject headings

## References

[1] Fukuda S, Takahashi T, Kumori K (2009). Idiopathic testicular infarction in a boy initially suspected to have acute epididymo-orchitis associated with mycoplasma infection and Henoch–Schönlein purpura. J Pediatr Urol.

[2] Baskin LS, Carroll PR, EV C, JW M (1990). Necrotising soft tissue infections of the perineum and genitalia: bacteriology, treatment and risk assessment. Br J Urol.

[3] Banyra O, Shulyak A (2012). Acute epididymo-orchitis: staging and treatment. Cent Eur J Urol.

[4] Ching-di Chang JLCLYCCHYLSNSK (2016). Acute Epididymo-orchitis–related global testicular infarction: clinical and ultrasound findings with an emphasis on the Juxta-epididymal string-of-bead sign. Ultrasound Q.

[5] Lefort C, Thoumas D, Badachi Y (2001). Ischemic orchiditis: review of 5 cases diagnosed by color Doppler ultrasonography. J Radiol.

[6] Sue SR, Pelucio M, Gibbs M (1998). Testicular infarction in a patient with epididymitis. Acad Emerg Med.

[7] Yusuf G, Sellars ME, Kooiman GG, Diaz-Cano S, Sidhu PS (2013). Global testicular infarction in the presence of epididymitis. J Ultrasound Med.

[8] Vordermark JS, Favila MQ (1982). Testicular necrosis: a preventable complication of epididymitis. J Urol.

[9] Pilatz A, Wagenlehner F, Bschleipfer T, et al. Acute epididymitis in ultrasound: Results of a prospective study withbaseline and follow-up investigations in 134 patients. Eur J Radiol. 2013;82(12). 10.1016/j.ejrad.2013.08.050. 10.1016/j.ejrad.2013.08.050 24094645

[10] Pilatz A, Hossain H, Kaiser R (2015). Acute epididymitis revisited: impact of molecular diagnostics on etiology and contemporary guideline recommendations. Eur Urol.

[11] Hourihane DO (1970). Infected infarcts of the testis: a study of 18 cases preceded by pyogenic epididymoorchitis. J Clin Pathol.

[12] PARR NJ, PRASAD BRP, HAYHURST V, McMILLAN A, LEEN CS, FOWLER JW (1993). Suppurative Epididymo-orchitis in young “high risk” patients-a new problem?. Br J Urol.

[13] Chia D, Penkoff P, Stanowski M, Beattie K, Wang AC (2016). Testicular infarction and rupture: an uncommon complication of epididymo-orchitis | Kopernio. J Surg Case Rep.

[14] Eisner DJ, Goldman SM, Petronis J, Millmond SH (1991). Bilateral testicular infarction caused by epididymitis. Am J Roentgenol.

[15] Rencken RK, DJ du P, De HLS. Venous infarction of the testis - a cause of non-response to conservative therapy in epididymo-orchitis. South African Med J. 78(9):337–8 https://www.ajol.info/index.php/samj/article/view/160180/149763. Accessed January 5, 2019. 2396157

[16] ERTC O, Kitson JL, Green B (1990). Venous infarction of the testis secondary to acute epididymitis. Br J Urol.

[17] Bird K, Rosenfield AT (1984). Testicular infarction secondary to acute inflammatory disease: demonstration by B-scan ultrasound. Radiology..

[18] Lu Y, Bhushan S, Tchatalbachev S (2013). Necrosis is the dominant cell death pathway in uropathogenic Escherichia coli elicited epididymo-orchitis and is responsible for damage of rat testis. PLoS One.

